# Draft Genome Sequence of the Spore-Forming Probiotic Strain *Bacillus coagulans* Unique IS-2

**DOI:** 10.1128/genomeA.00225-16

**Published:** 2016-04-21

**Authors:** Aditya Upadrasta, Swetha Pitta, Ratna Sudha Madempudi

**Affiliations:** Centre for Research and Development, Unique Biotech Limited, Hyderabad, India

## Abstract

*Bacillus coagulans* Unique IS-2 is a potential spore-forming probiotic that is commercially available on the market. The draft genome sequence presented here provides deep insight into the beneficial features of this strain for its safe use as a probiotic for various human and animal health applications.

## GENOME ANNOUNCEMENT

Among the lactic acid bacteria (LAB), spore formers have gained much attention due to their ability to withstand adverse conditions during production, downstream processing, storage, and gastric transit (1, 2). Their high survival rates under hostile conditions make them the primary choice of probiotic candidates to consume either as oral medications or for incorporation into functional foods. *Bacillus coagulans* Unique IS-2 has been isolated from healthy human feces (3) and has obtained a “no objection” rating from the U.S. FDA to its generally regarded as safe (GRAS) status. *B. coagulans* Unique IS-2 is a nontoxic, safe spore-forming probiotic, which has shown therapeutic effect in clinical disorders, like hypercholesterolemia (4), bacterial vaginosis (5), and acute diarrhea (6), and in the treatment of functional abdominal pain in children (7). Furthermore, *in vitro* studies have indicated the efficacy of this strain to withstand high temperatures, its gastric tolerance, and its ability to metabolize an array of sugars (3).

Here, we report the draft genome sequence of *B. coagulans* Unique IS-2 in order to unravel the genetic blueprint that confers its probiotic traits and safe use.

Whole-genome sequencing was performed using the Illumina MiSeq platform at the Institute of Microbial Technology (IMTECH), Chandigarh, India. The Illumina sequencing libraries were prepared using the Nextera XT sample preparation kit with dual indexing adaptors. A total of 971,128,104 paired-end reads were obtained, providing 282-fold coverage depth of the genome. Among them, 43,446,692 Illumina reads were *de novo* assembled using CLC Genomics Workbench 7.5. The assembled genome sequence was annotated by the RAST annotation pipeline Web server (8, 9) and by NCBI Prokaryotic Genome Annotation Pipeline (PGAP) version 2.9. rRNAs and tRNAs were annotated using RNAmmer (10) and tRNAscan-SE (11), respectively. The genome analysis was performed using the Artemis genome viewer (12).

The draft genome sequence assembled to 143 contigs composed of 3,446,692 bp, with an average G+C content of 46.4%. The genome contains a total of 3,870 protein-coding sequences (CDSs), including 80 tRNAs and 3 rRNAs, with a coding percentage of 84.2% and 1.12 gene density per kilobase. The genome consists of clustered regularly interspaced short palindromic repeat (CRISPR) cassettes and two putative bacteriocin operons.

Furthermore, SEED metabolic analysis revealed 2,638 functional genes (434 subsystems), of which the highest number accounted for central carbohydrate metabolism (501 genes, including xylose, sucrose, maltose, and maltodextrin and chitin utilization), amino acids and derivatives (361 genes), protein metabolism (240 genes), cofactors and vitamins (197 genes), RNA metabolism (142 genes), sporulation and dormancy (92 genes), and stress response (84 genes).

Generation of the complete genetic blueprint of *B. coagulans* Unique IS-2 revealed many interesting probiotic traits and shed light on the molecular mechanism at the base of its probiotic and beneficial properties, widening its horizons as a safe probiotic for extensive use in food and pharmaceutical preparations.

### Nucleotide sequence accession numbers.

This whole-genome shotgun project has been deposited at DDBJ/ENA/GenBank under the accession no. JZDH00000000. The version described in this paper is version JZDH01000000.

## References

[1] Cutting SM 2011 *Bacillus* probiotics. Food Microbiol 28:214–220. doi:10.1016/j.fm.2010.03.007. 21315976

[2] Sanders ME, Morelli L, Tompkins TA 2003 Sporeformers as human probiotics: *Bacillus*, *Sporolactobacillus*, and *Brevibacillus*. Compr Rev Food Sci Food Saf 2:101–110. doi:10.1111/j.1541-4337.2003.tb00017.x. 33451235

[3] Sudha R, Chauhan P, Dixit K, Babu SM, Jamil K 2010 Molecular typing and probiotic attributes of a new strain of *Bacillus coagulans* unique IS-2: a potential biotherapeutic agent. Genet Eng Biotechnol J 7:1–20.

[4] Sudha MR, Radkar N, Maurya A 2011 Effect of supplementation of probiotic *Bacillus coagulans* unique IS-2 (ATCC PAT-11748) on hypercholesterolemic subjects: a clinical study. Int J Probiotics Prebiotics 6:89–93.

[5] Ratna Sudha MR, Yelikar KA, Deshpande S 2012 Clinical study of *Bacillus coagulans* unique IS-2 (ATCC PTA-11748) in the treatment of patients with bacterial vaginosis. Indian J Microbiol 52:396–399. doi:10.1007/s12088-011-0233-z. 23997330 PMC3460128

[6] Sudha RM, Bhonagiri S 2012 Efficacy of *Bacillus coagulans* strain Unique IS-2 in the treatment of patients with acute diarrhea. Int J Probiotics Prebiotics 7:33–37.

[7] Saneian H, Pourmoghaddas Z, Roohafza H, Gholamrezaei A 2015 Synbiotic containing *Bacillus coagulans* and fructo-oligosaccharides for functional abdominal pain in children. Gastroenterol Hepatol Bed Bench 8:56–65. 25584177 PMC4285933

[8] Aziz RK, Bartels D, Best AA, DeJongh M, Disz T, Edwards RA, Formsma K, Gerdes S, Glass EM, Kubal M, Meyer F, Olsen GJ, Olson R, Osterman AL, Overbeek RA, McNeil LK, Paarmann D, Paczian T, Parrello B, Pusch GD, Reich C, Stevens R, Vassieva O, Vonstein V, Wilke A, Zagnitko O 2008 The RAST server: Rapid Annotations using Subsystems Technology. BMC Genomics 9:75. doi:10.1186/1471-2164-9-75. 18261238 PMC2265698

[9] Overbeek R, Olson R, Pusch GD, Olsen GJ, Davis JJ, Disz T, Edwards RA, Gerdes S, Parrello B, Shukla M, Vonstein V, Wattam AR, Xia F, Stevens R 2014 The SEED and the rapid annotation of microbial genomes using subsystems technology (RAST). Nucleic Acids Res 42:D206–D214. doi:10.1093/nar/gkt1226. 24293654 PMC3965101

[10] Lagesen K, Hallin P, Rødland EA, Stærfeldt H-H, Rognes T, Ussery DW 2007 RNAmmer: consistent and rapid annotation of ribosomal RNA genes. Nucleic Acids Res 35:3100–3108. doi:10.1093/nar/gkm160. 17452365 PMC1888812

[11] Lowe TM, Eddy SR 1997 tRNAscan-SE: a program for improved detection of transfer RNA genes in genomic sequence. Nucleic Acids Res 25:955–964. doi:10.1093/nar/25.5.0955. 9023104 PMC146525

[12] Rutherford K, Parkhill J, Crook J, Horsnell T, Rice P, Rajandream M-Al, Barrell B 2000 Artemis: sequence visualization and annotation. Bioinformatics 16:944–945. doi:10.1093/bioinformatics/16.10.944. 11120685

